# On the relevance of the alpha frequency oscillation’s small-world network architecture for cognitive flexibility

**DOI:** 10.1038/s41598-017-14490-x

**Published:** 2017-10-24

**Authors:** Nicole Wolff, Nicolas Zink, Ann-Kathrin Stock, Christian Beste

**Affiliations:** 1 Cognitive Neurophysiology, Department of Child and Adolescent Psychiatry, Faculty of Medicine, TU, Dresden, Germany; 2 Experimental Neurobiology, National Institute of Mental Health, Klecany, Czech Republic

## Abstract

Cognitive flexibility is a major requirement for successful behavior. nNeural oscillations in the alpha frequency band were repeatedly associated with cognitive flexibility in task-switching paradigms. Alpha frequencies are modulated by working memory load and are used to process information during task switching, however we do not know how this oscillatory network communication is modulated. In order to understand the mechanisms that drive cognitive flexibility, ERPs, oscillatory power and how the communication within these networks is organized are of importance. The EEG data show that during phases reflecting preparatory processes to pre-activate task sets, alpha oscillatory power but not the small world properties of the alpha network architecture was modulated. During the switching only the N2 ERP component showed clear modulations. After the response, alpha oscillatory power reinstates and therefore seems to be important to deactivate or maintain the previous task set. For these reactive control processes the network architecture in terms of small-world properties is modulated. Effects of memory load on small-world aspects were seen in repetition trials, where small-world properties were higher when memory processes were relevant. These results suggest that the alpha oscillatory network becomes more small-world-like when reactive control processes during task switching are less complex.

## Introduction

Cognitive flexibility is a major requirement for successful goal-directed behavior in real life situations and has been subject to intensive research in the past decades^1–3^. It depends on dedicated control mechanisms associated with prefrontal cortical structures^4^. The neural mechanisms underlying cognitive flexibility are often examined in task switching paradigms^5^ where switches between responses lead to an increase in processing times as compared to non-switch task response times; i.e. switch costs^5^. Several mechanisms have been suggested to explain switch cost (or residual switch costs), which are thought to reflect effects of past experience^6^. These possible mechanisms include shifts of attention between perceptual and conceptual elements, retrieving goals and condition-action rules from working memory or activation of relevant task sets and inhibition of irrelevant task sets^2,5,7^.

It has been shown that task switching processes are impaired when task switches need to be triggered by working memory processes^7–10^: Task switching reflects a process, which evolves a conflict in the “central executive” and takes more time than task repetition. The difference between task switching and repetition is depicted in switch costs, which reflects the time required to reconfigure the cognitive system. Processes that are involved in the reconfiguration and thus underlying mechanisms of switch costs are discussed controversial^11,12^. While, some accounts assume that reconfiguration only involves processes that operate on working memory i.e.^13,14^, others, argue that reconfiguration involves processes outside of working memory i.e.^15^. During switching tasks, in which external cues are absent, subjects are assumed to use internal prompts such as inner speech which help to retrieve currently relevant response sets^16,17^ while flexibly reacting on randomly alternating visual presented targets. Thus the need for concurrent articulation, while simultaneously reacting flexible on changing conditions are two processes, that likely interfere with each other and demand the central executive, which is a component of working memory^18^. Thus, in line with other studies where comparable^19–21^ or identical^7,10^ paradigms were used, we argue that during cue-based as compared to memory-based trials, reduced amounts of working memory are present.

The degree of such switch costs is affected by a number of factors^2^. One of these factors refers to the role of preparatory processes. Preparatory processes have been shown to reduce switch costs^2,22,23^, because proactive control processes may prepare the system for an upcoming need for goal-appropriate control of behaviour and activate necessary task sets to perform switching^24^. On a neurophysiological level, such proactive control processes have been shown to be associated with modulations in alpha frequency oscillations^24–28^. However, aside proactive control processes, also reactive processes play a role in task switching^24^. Since past experience and previous task sets (response rules) stored in working memory strongly shapes switching processes^2,6,29^ reactive control processes are useful to inhibit interferences arising from previous tasks^30–33^. Interestingly, alpha frequency oscillations have also been shown to be important in inhibitory control processes regulating access of information of a knowledge system and working memory^34,35^, which is important for task set activation and switching^31–33^. The described inhibition processes are highly relevant to task switching, since higher switch costs are accompanied by higher inhibitory control processes^30,36^. In addition alpha frequencies have also been shown to increase with working memory load during a retention interval^37^ and are modulated by the degree of working memory based interference^38^. Moreover alpha frequencies have been shown to be evident during preparation and post-target intervals during task switching which was linked to general task performance^39^. With respect to network communication within frequency bands, it has been shown that local clustering and global efficiency of this communication can be attributed to distinct frequency bands. Small world properties that support working memory, was observed to be most pronounced in the alpha frequency band^40^. However, the way those networks communicate via alpha oscillations remains elusive. The aim of this study is therefore to investigate i) how alpha oscillatory communication between networks is organized before (preparatory processes) and after task switching (reconfiguration processes) and ii) how these processes are modulated by different demands on cognitive flexibility (i.e. increased working memory interference). Thus, in order to elucidate the alpha oscillatory network mechanisms that drive cognitive flexibility, we examined the so called “small world” network architecture of alpha oscillations during task switching. Networks showing small-world properties show dense local interconnectivity and short average path length linking nodes in a short and efficient way, which is essential for efficient separation and functional integration of information especially during cognitive flexibility^41–43^. This network perspective is important, because executive functions and mechanisms of cognitive flexibility need to be understood in terms of dynamics in a network^4^ and oscillations in the alpha band have been suggested to coordinate top-down control processes and large-scale communication within and between neural networks^44,45^. When analyzed with graph-theoretical concepts, most real-world networks develop “small world” properties^41–43,46^. Small-world networks show a dense local interconnectivity and short path length linking individual network nodes in a short and efficient way^46^. This, together with a high density of connections between nearest neighbors is necessary for efficient separation and functional integration of information^41,42,47^. Since alpha oscillations are important for the coordination of large-scale communication within and between neural networks^44,45^ and seem to play a central role in mechanisms important for task switching processes (see above), the examination of the actual networks architecture in terms of small-world properties is particularly important.

It is therefore likely that there are considerable differences in power and small-world network architecture of alpha frequency oscillations between task switching using memory-based or cue-based processes. Since both, preparatory processes and processes of inhibition or deactivation of applied task sets are known to modulate i) demands on task switching processes and ii) alpha oscillations (see above), goal of this study is to examine whether there are differential effects of preparatory, inhibition and reconfiguration processes in addition to varying demands on memory processes during task switching on small-world network properties of alpha oscillations. However, a small world network has been suggested to be important for an efficient separation and functional integration of information^41,42,47^. Yet, during task switching, the integration of information is not straightforward and efficient. This is because responses have to be dynamically selected and reconfigured^5^, goals and condition-action rules have to be determined and retrieved from working memory, and task sets have to become inhibited or deactivated^1,2^. If this is the case it is also possible that under switching conditions the alpha frequency network shows lower small-world network properties compared to response repetition conditions. Since working memory load has been shown to compromise task switching performance^7–10^, these effects may be stronger when working memory processes trigger task switching processes.

In addition to alpha frequency related power and network patterns we perform a standard analysis using event-related potentials (ERPs). Since it has been shown that the P3 and N2 ERP-components reflect mechanisms of task set activation and or in processes reflecting the resolution of conflict (or task-set inertia), respectively^48–51^, we focus on the analysis of these two ERP-components (for analysis of P1 and N1 refer to supplemental material). Both ERP-components have been shown to be modulated in the paradigm applied in this task^7–10^.

## Results

### Behavioral data

#### Reaction times (RTs)

**Figure 1:**
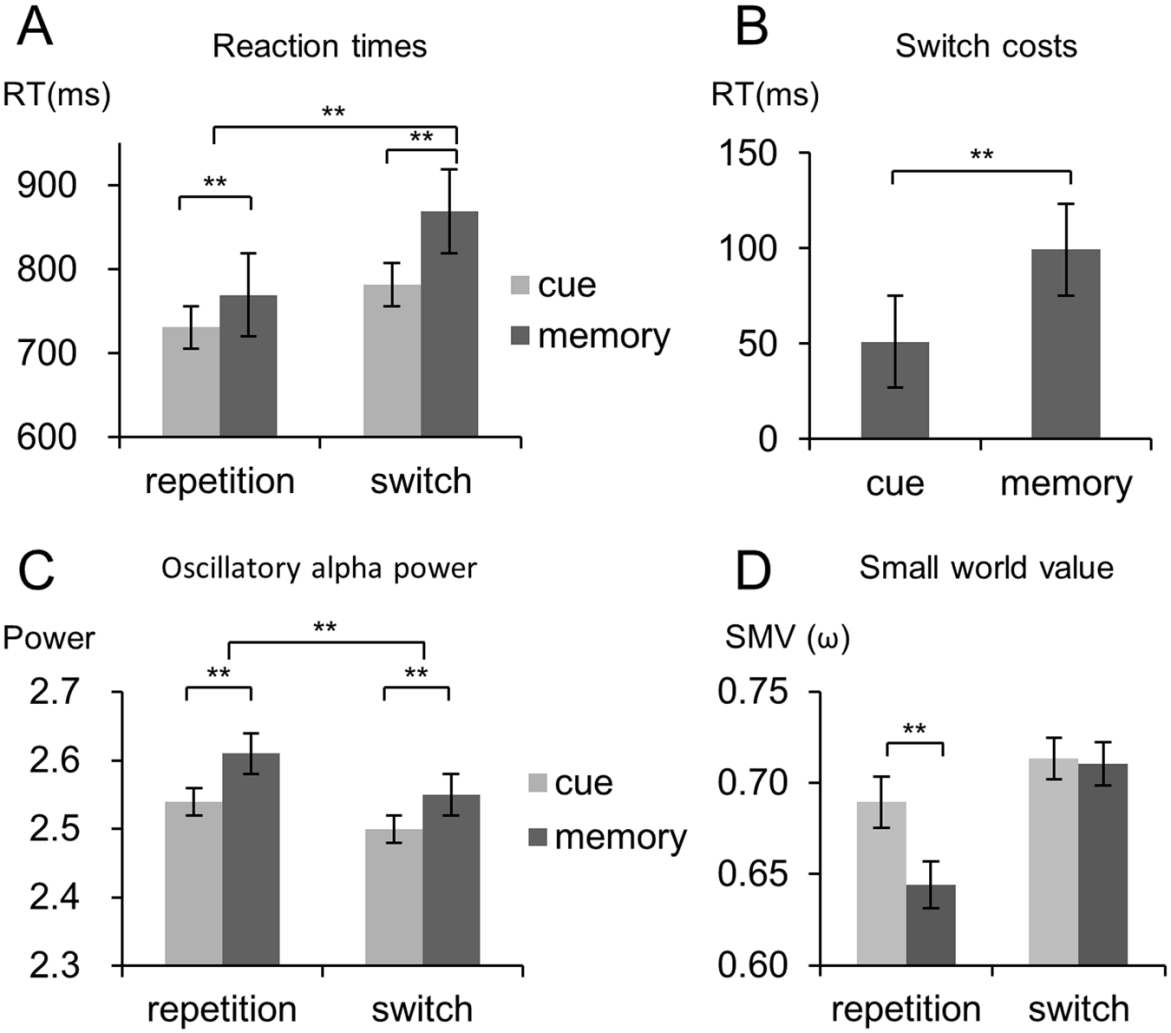
(**A**) Switching costs in milliseconds for the cue-based and the memory-based block. (**B**) Reaction times (RTs) showing the mean and standard error of the mean in the different experimental conditions. (**C**) Significant interaction found for the small world values (SWVs) (y-axis) for cue and memory-based trials in the repetition and the switching condition. The mean and standard error of the mean are given. (**D**) The oscillatory alpha power showing the mean and standard error of the mean in the different experimental conditions.

A repeated measures ANOVA on mean RTs with the within-subject factors “condition” and “block” yielded the following significant main effects: “block” (*F*[1,54] = 38.14 *p* < 0.001, *η* ^2^_*p*_ = 0.381), with faster RTs during cue- (749 ms ± 21.82) than memory-based blocks (813 ms ± 21.85) as well as “condition” (*F*[1,54] = 102.09 *p* < 0.001, *η* ^2^ = 0.622), with faster RTs during task repetition (745 ms ± 19) than task switching (818 ms ± 23). These effects were further specified by the significant interaction “condition x block” (*F*[1,54] = 25.64 *p* < 0.001, *η* ^2^_*p*_ = 0.293), which is shown in Fig. 1A.

Post-hoc paired-t-tests revealed both, significantly (*t* [54] = 5.72, *p* < 0.001) increased RTs during cue-based (773 ms ± 24.04) switching vs. (726 ms ± 20.20) repetition trials as well as a significant effect (*t*[54] = 10.42, *p* < 0.001) during memory-based switching (863 ms ± 23.57) vs repetition (763 ms ± 21.08) trials. However, effects were larger during memory-based switching vs. repetition conditions than cue-based switching vs. repetition condition, which is also shown in the increased residual switch costs during memory-based bocks.

#### Residual switch costs

Residual switch costs were obtained for correct trials by subtracting response times (RTs) during task repetition from RTs during task switching (cue-based SC = RT cue-based-switching − RT cue-based-repetition; memory-based SC = RT memory-based-switching − RT memory-based-repetition). A repeated measures ANOVA using the within-subject factor “block (cue- vs. memory-based)” revealed a main effect of “block” (*F*(1,54) = 25.64, *p* < 0.001, *η* ^2^_*p*_ = 0.293), indicating significantly increased residual switch costs during memory- (99 ms ± 9.55) as compared to cue-based (47 ms ± 8.23) blocks Fig. 1B.

#### Accuracy

A repeated measures ANOVA on accuracy (percentages of hits) using the within-subject factors “condition” and “block” yielded two significant main effects: The main effect “block” (*F*[1,54] = 8.45, *p* = 0.005, *η* ^2^_*p*_ = 0.120) showed higher accuracy during the “cue-“ (93.77% ± 0.43) as compared to “memory-based blocks” (91.90% ± 0.67). The main effect “condition” (*F*[1,54] = 37.96 *p* < 0.001, *η* ^2^_*p*_ = 0.380) showed higher accuracy during task repetition (94.10% ± 0.41) than during task switching (91.57% ± 0.59). The interaction between both factors was not significant (*p* > 0.3). Together with the RT data, the results show that there was no speed-accuracy trade-off.

#### Neurophysiological Results

Analysis of the P1 and N1 ERP-component can be found in the supplemental material.

**Figure 2:**
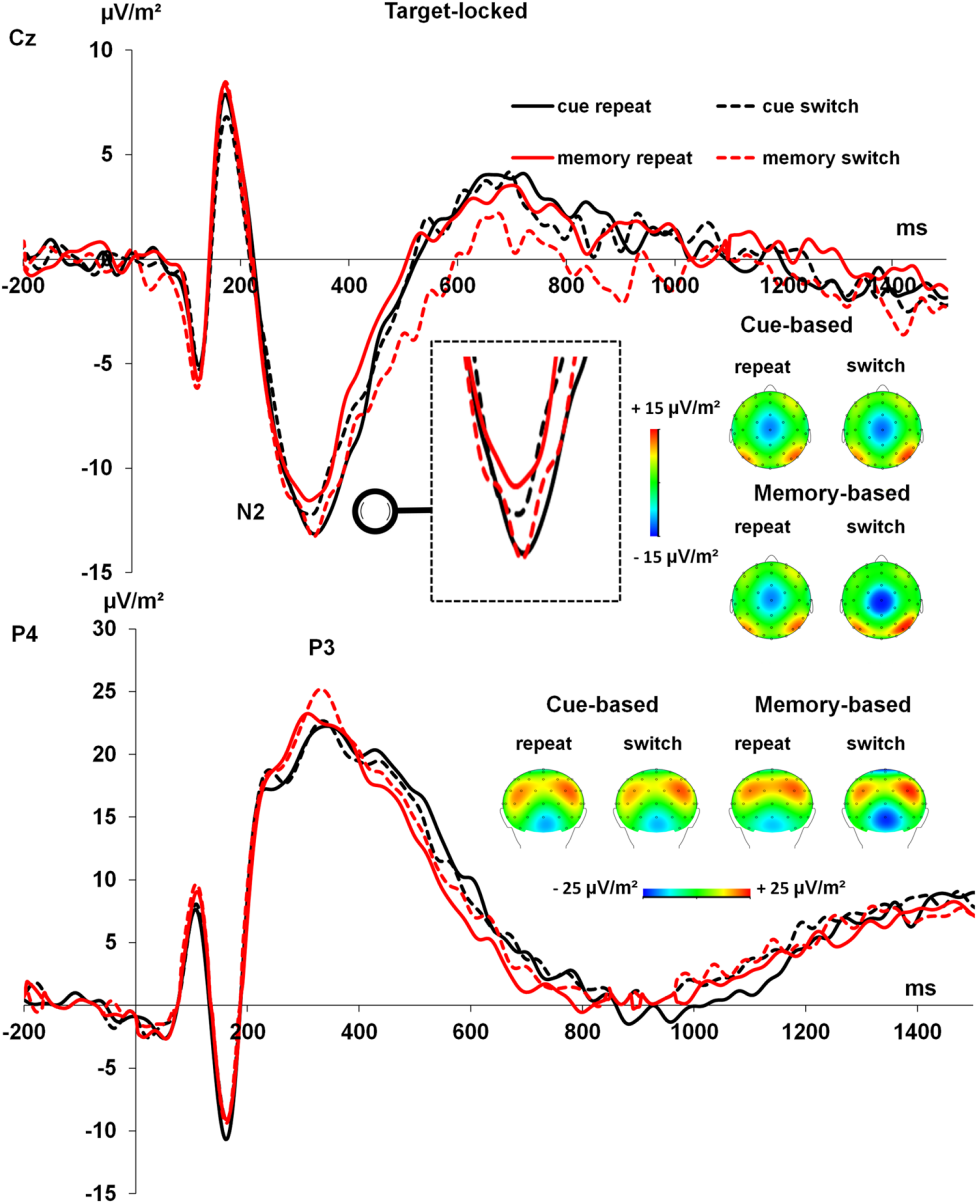
The target-locked N2 and P3 ERP-components are shown using a baseline from −200 ms to zero (i.e. time point of target presentation). The N2 and P3 ERP-components on the target are shown at electrodes Cz and P4, respectively. Time point zero denotes the time point of target stimulus presentation. The cue is presented ~1300 ms before the target. Cue-based conditions are given in black lines, memory-based conditions are given in red lines. Repetitions are shown in solid lines, switches are shown in dashed lines. The scalp topography plots show the distributions of potentials across the scalp at the time point of the peak of each ERP-component. In the topography plots red colours denote positive scalp potentials, blue denotes negative scalp potentials. Data were down-sampled to 256 Hz and filtered with a band-pass filter from 0.5 to 25 Hz, with a slope of 48 dB/oct each.

Target-locked N2 and P3 ERP-components were analysed using a repeated measures ANOVAs with the within-subject factors “condition” and “block”. The respective ERP components are shown in Fig. 2.

*N*2: The ANOVA on N2 mean amplitudes at electrode Cz revealed a significant interaction of “condition x block” (*F*[1,54] = 4.33, *p* = 0.042, *η* ^2^_*p*_ = 0.074). Post-hoc paired t-tests showed no significant difference between switching (−13.67 µV/m^2^ ± 1.62) and repetition (−14.02 µV/m^2^ ± 1.58) trials in cue-based blocks (*t*[54] = 0.440, *p* = 0.662), but there were significantly increased (more negative) N2 amplitudes during switching (−14.56 µV/m^2^ ± 1.39) vs. repetition trials (−12.43 µV/m^2^ ± 1.27) in the memory-based block (*t*[54] = −2.09, *p* = 0.041). No further effects were significant (all *p* > 0.206).

*P3*: An ANOVA on the P3 amplitudes at P4 revealed significant main effects of “block” (*F*[1,54] = 4.77, *p* = 0.033, *η* ^2^_*p*_ = 0.081) with increased (more positive) amplitudes in the memory- (22.37 µV/m^2^ ± 2.14) as compared to the cue-based block (20.48 µV/m^2^ ± 2.04) and “condition” (*F*[1,54] = 4.77, *p* = 0.033, *η* ^*2*^_*p*_ = 0.081), showing increased amplitudes during task switching (22.47 µV/m^2^ ± 2.10) vs. task repetition (20.38 µV/m^2^ ± 2.05). Importantly, both main effects were further specified by the significant interaction of “block x condition” (*F*[1,54] = 4.75, *p* = 0.034, *η* ^2^_*p*_ = 0.081). Post-hoc paired t-tests showing no significant difference between switching (20.76 µV/m^2^ ± 2.12) and repetition (20.19 µV/m^2^ ± 2.06) trials in cue-based blocks (*t*[54] = 0.609, *p* = 0.545). However, the P3 amplitudes were significantly larger during switching (24.18 µV/m^2^ ± 2.24) vs. repetition (20.56 µV/m^2^ ± 2.16) trials in memory-based blocks (*t*[54] = 3.37, *p* < 0.001). This interaction is line with the interaction observed for the behavioral data. There were no latency effects for the N2 and P3 on target stimuli (all *p* > 0.3).

#### Alpha-band oscillations

**Figure 3:**
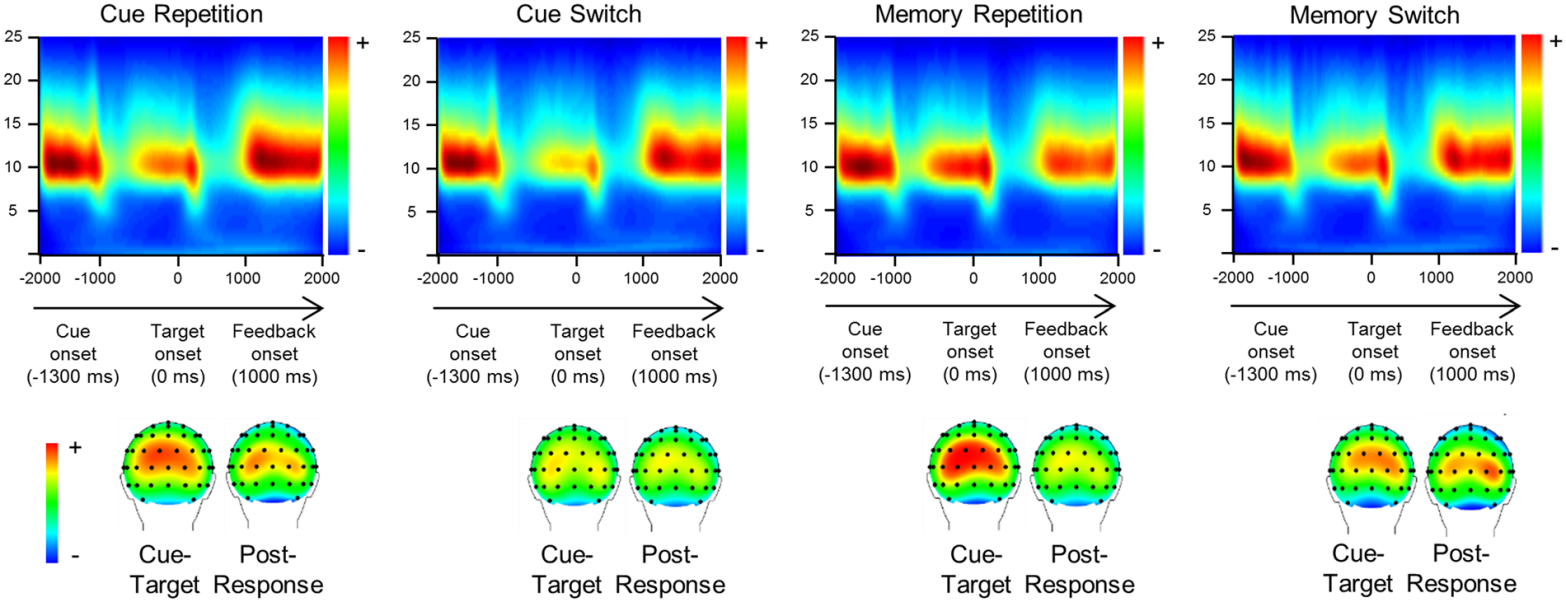
Results from the time frequency decomposition. Clear alpha band activity can be seen in each plot, which shows the average alpha band power across electrodes P7, P8, O1, O2, PO1, PO2. The upper row shows the repetition condition, the lower row the switching condition. The left column shows the cue-based block, the right column the memory-based block. The x-axis for each time frequency plot shows the time in milliseconds, the y-axis the frequency. The colour coding reflects the strength of the alpha band power. Time point zero denotes the time point of target stimulus presentation. The cue is presented ~1300 ms before the target. The scalp topography plots are given for the cue-target interval, as well as for the interval 800 ms to 1500 ms after target presentation.

The time-frequency plots showing alpha band power (ABP) are given in Fig. 3 for each experimental condition. The time-frequency plots show the alpha band activity averaged across electrodes P7, P8, O1, O2, PO1, PO2, because these electrodes were identified in the electrode selection step (refer methods section) and there was no main or interaction effect “electrode”.

*Post-Cue Processes of alpha-band oscillations (−1300–0* *ms):* For the cue-target interval, the mixed model ANOVA on the cue-target ABP with the within-subject factors “condition” (repetition switching,), “block” (cue-based, memory-based) and “electrode” (P7, P8, O1, O2, PO1, PO2) revealed significant main effects of “block” (*F*[1,54] = 11.91, *p* < 0.001, *η* ^2^_*p*_ = 0.172), with increased ABP during memory- (2.58 ± 0.05) vs. cue-based blocks (2.52 ± 0.05) as well as a significant main effect of “condition” (*F*[1,54] = 17.43, *p* < 0.001, *η* ^2^_*p*_ = 0.237) with increased ABP during repetition (2.57 ± 0.05) vs. switching (2.53 ± 0.05) conditions. In addition a significant interaction of “condition x block” (*F*[1,54] = 7.01, *p* = 0.010, *η* ^2^_*p*_ = 0.112), further specifying the main effects. Post-hoc paired t-tests revealed significantly increased ABP during cue-based repetition (2.54 ± 0.05) vs. switching (2.50 ± 0.05) trials (*t*[54] = 2.78, *p* = 0.007) as well as during memory-based repetition (2.61 ± 0.06) vs. switching (2.55 ± 0.05) trials (*t*[54] = 4.00, *p* < 0.001). Similarly to the behavioral performance effects were larger during memory-based repetition vs. switching condition than in cue-based repetition vs. switching condition. No further main effect or interaction was significant (all *p* > 0.081).

*Post Target processes of alpha-band oscillations (800–1500* *ms):* For the interval from 800 to 1500 ms post target, the mixed model ANOVA on the post-target alpha band power with the within-subject factors “condition” (repetition, switching), “block” (cue-based, memory-based) and “electrode” revealed no significant effect (all *p* > 0.054), but a trend for “block” (F(1,57) = 3.871, p = 0.054, *η* ^2^_*p*_ = 0.064*)*, indicating increased ABP during cue- vs memory-based blocks. Importantly, due to the long and jittered response-cue interval (RCI) (~2000 ms), the analyzed time interval does not simply reflect preparatory processes of the upcoming cue-stimuli.

#### Analysis of the alpha band small world network architecture

*Post-Cue processes of alpha-band oscillations (−1300–0* *ms):* The analysis of the small world value (SWV) in the cue-target interval revealed no main or interaction effects regarding SWV (all *p* > 0.25).

**Figure 4:**
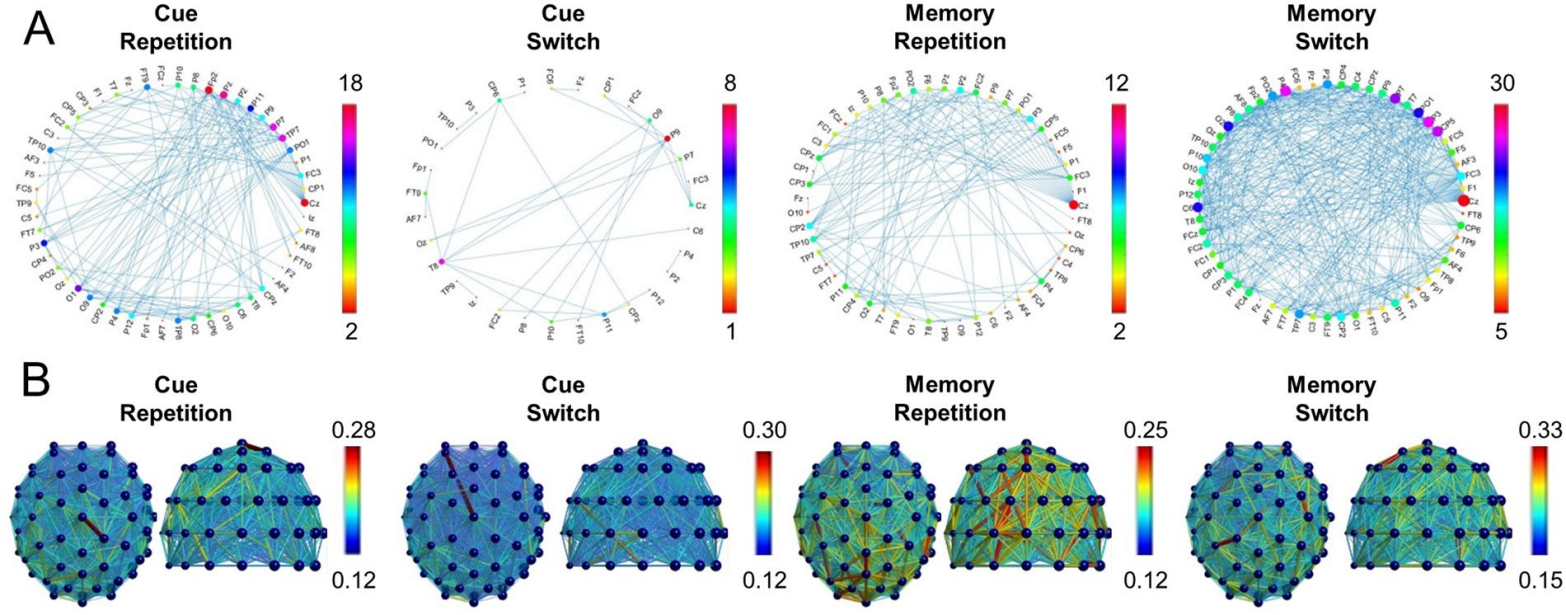
Results from the connectivity analysis. (**A**) Alpha oscillation based networks for the different experimental conditions (cue-based repetition, cue-based switch, memory-based repetition, memory-based switch). The imaginary part of the coherence is plotted as edges between the electrodes (nodes). The colour bar denotes the number of connections from one electrode to other electrodes. (**B**) Alpha oscillation based networks between the EEG electrodes for the different experimental conditions (cue-based repetition, cue-based switch, memory-based repetition, memory-based switch) plotted from the back and top view on the scalp. The colour bar denotes the strength of the imaginary part of the coherence.

*Post-Target processes of alpha-band oscillations (800–1500* *ms):* The analysis of the SWV of alpha frequency activity in the time range from 800 ms to 1500 ms after target stimulus revealed effects. The connectivity pattern between electrodes for all experimental conditions is shown in Fig. 4(A and B). The analysis of the SWV revealed the following: The was a main effect “block” (*F*[1,54] = 4.08; *p* = 0.048; *η* ^2^_*p*_ = 0.068) showing that the SWV was lower in the memory-based block (0.67 ± 0.01) than in the cue-based block (0.70 ± 0.01). The main effect “condition” (*F*[1,54] = 26.13; *p* < 0.001; *η* ^2^_*p*_ = 0.318) showed that the SWV was smaller during repetition (0.66 ± 0.01) than switching trials (0.71 ± 0.009). There was an interaction “block x condition” (*F*[1,54] = 5.27; *p* = 0.025; *η* ^2^_*p*_ = 0.086), which is shown in Fig. 1C. Post-hoc tests showed that for switching trials no difference between the memory-based and the cue-based block was evident (*t*[54] = 0.20; *p* > 0.4). In the repetition trials, the SWV was larger in the cue-based condition (0.68 ± 0.01) than in the memory-based condition (0.64 ± 0.01) (*t*[56] = 2.98; *p* = 0.002).

It is important that within the spectrum of maximum smallworldness (ω = 0) and maximum randomness (ω = 1) the genereal SWV is on average (ω = 0.67) more random than smallworldish.

## Discussion

In the current study we focused on how alpha oscillations are modulated by working memory load during task switching and how oscillatory network communication within thus frequency range is modulated in order to process information. Therefore, alpha frequency oscillations and the network architecture before and after task switching processes as well as ERP processes were examined via a memory- and cue-based task switching paradigm.

### Behavioral, ERP and alpha band power data

The behavioral data show common differential effects of the cue- and memory-based block during switching^7,9^. Residual switch costs in response times (RTs) were generally higher in the memory-based block than in cue-based block. This effect was due to strong effects of the block on switching trials, where RTs were longer in the memory-based block than in the cue-based block. This possibly reflects an effect of modulated updating processes of internal representations and task sets for response selection. Underlining this interpretation an analysis of ERPs showed that the N2 and P3 ERP components reflected the interaction observed at the behavioral level. The higher N2 on switching trials in the memory-based block well reflects increased processes, which are related to the resolution of conflict (or task-set inertia, reflected by the N2)^48–51^. The finding of no similar modulations on cue-based switching trials underlines that cuing reduced demands on task switching processes, which is well in line with the literature^2,22,23^. Opposed to the N2 component, the P3 component did not well reflect the behavioural data. The P3 revealed a parietal maximum, but showed larger amplitudes in switching than in repetition trials, opposed to smaller amplitudes as frequently found^48–55^. It has been shown that mechanisms of working memory processes are reflected by the P3 ERP-component^56–59^. This may have led to the unusual increase in P3 amplitudes on switching trials. It can also not be ruled that this odd effect reflects a volume conduction effect of processes found for the N2 component.

The time-frequency data show a general increase in alpha frequency activity before and after task switching, in the post-cue (cue-target) interval and in the time period immediately following the response (~700 and 800 ms); i.e. between 800 and 1500 ms after both target presentation and response (see Fig. 3). These modulations can be disentangled as follows: within the cue-target interval the observed alpha oscillations likely reflect preparatory processes, as described previously^24–27^. The data shows that during repetition trials alpha power was stronger than during switching trials and that this effect was further amplified in the memory-based block. This is well in line with findings showing that alpha oscillations increase with working memory load^60,61^. The results suggest that proactive control processes exerted are intensified in the memory-based block, possibly because more memory-load increases demands on working memory processes.

Interestingly, we observed no modulatory effects in the alpha oscillatory power in the time range from 800–1500 ms. Alpha oscillations have been suggested to reflect inhibitory control processes regulating access of information of a knowledge system and working memory^34,35^, which is important for task set activation and switching^30–33^. Therefore, the data shows that these reactive control mechanisms are not coded in the power of the signal. Yet, the analysis of the small-world data suggests that modulations were observable at the level of the alpha network in terms of small-world properties.

### Alpha band small world network analysis

The analysis of the SWV providing information about the small-world property of the network revealed an interaction “condition x block” in the period from 800 to 1500 ms after target presentation (i.e., where no modulations in alpha power were evident). As stated above, this second interval of strong alpha frequency activity immediately follows the executed responses, since the mean RT was between ~700 and 800 ms. Therefore, this time interval likely reflects reactive control processes. Importantly, due to the long and jittered response-cue interval (RCI) (~2000 ms), the analyzed time interval does not simply reflect preparatory processes of the upcoming cue-stimuli.

In the time interval from 800 to 1500 ms, alpha network properties in general had more properties of transmitting information effectively (more random network properties) than being very smallworldish and they are even further away from being specialized (more regular/lattices). In particular, the SWV was higher in switching than repetition trials in both, the memory-based and the cue-based condition. This suggests that once the response has been executed and the response selection process has been finished, the degree of efficient separation and functional integration of information on a network level becomes important^41,42,47^. After responses in switching trials, the SWV was larger than after responses in repetition trials; which means that the networks becomes more complex (random) in switching than repetition trials. Alpha oscillations have been suggested to coordinate top-down control processes^44,45^ and reflect inhibitory mechanisms to control access to task-relevant information^35^. During switching trials such control processes are useful to inhibit interferences arising from previous tasks^30–33^. Because differences in the alpha SWV were evident after the execution of the response, it is possible that this reflects inhibition processes of the previous task set. The more random network architecture may convey more degrees of freedom for such processes to unfold and may make it easier to coordinate the necessary neural processes. Notably, these processes are not different between the memory-based and the cue-based block, suggesting that memory load in conditions requiring cognitive flexibility is not important to consider.

However, opposed to switching trials, strong effects between the cue-based and the memory-based blocks were observed for alpha oscillations after responses in repetition trials. This suggests that mechanisms related to switching are more powerful to alter alpha oscillatory network properties than it is the case for working memory load. The SWV was smaller in the memory- than in the cue-based block; i.e. in the memory-based block the alpha frequency networks showed more small-world properties than in cue-based block. This may be explained as follows: Task sets are stored in working memory^62^ and once a response has been executed, it increases task performance when this task set needs to be inhibited or deactivated^30^ - a function of alpha frequency oscillations^35^. However, in the memory-based block access to the task sets stored in working memory may be easier and more direct than in the cue-based block, because the cognitive operations have already been triggered from working memory. This may make it easier to integrate information and to coordinate top-down processes regulating task set inhibition and may reduce demands on the network to flexibly integrate information. Therefore, the alpha oscillation network is less complex (random) and shows stronger small-world properties.

Of special interest is the comparison with the result pattern obtained in the N2 ERP-component data. As discussed above, the N2 ERP data well reflects the behavioral data and is likely to reflect processes of task set activation. Alpha frequency oscillations and differences in the small-world network architecture may be interpreted as reflecting processes related to the inhibition of a previous task set (see above). Notably, in the time period of the N2 ERP, the time frequency plots show that no strong alpha activity was evident. It is therefore possible that the N2 ERP and alpha frequency oscillations reflect complementary processes related to the processing of task sets. It seems that the alpha network architecture is important for task set inhibition processes (reactive control processes), but not for preparation and activation processes; i.e. proactive control processes during task switching. Underlining this interpretation, there were no differences in small-world network architecture in alpha oscillations in the cue-target interval, in which proactive control processes preparing the system for an upcoming need for goal-appropriate are evident^24–27^.

## Conclusions

The study focused on the role of alpha frequency oscillations and its network architecture during memory-based and cue-based task switching processes. The results show that alpha oscillations are modulated during phases of proactive control processes, likely reflecting processes to pre-activate task sets, but these processes did not affect the network architecture in terms of small-world properties. Alpha oscillations are not modulated during the switching process per se, in which only the N2 and ERP-component showed clear modulations. Alpha oscillations reinstate after the response and therefore seem to be important to maintain or deactivate the previous task set. For these reactive control processes the network architecture in terms of small-world properties is important. Generally, after task switches the network showed less small-world properties. Effects of memory load on small-world aspects were seen in repetition trials, where the network showed higher small-world properties when memory processes were relevant. These results suggest that the involved alpha oscillation network, had more properties of transmitting information effectively than being very smallworldish or specialized. Moreover, the involved alpha oscillation network becomes more small-world-like when reactive control processes during task switching are less complex. It is possible that small-world like network properties (i.e. higher degrees of freedom to organize network entities) helps to accomplish reactive control processes during task switching.

## Materials and Methods

### Participants

A total of n = 55 healthy young participants (24 females) between 18 and 30 years (*M* = 23.75 ± 0.49) took part in the experiment. All participants were right handed, had normal or corrected-to-normal vision, were free of medication and reported no psychiatric or neurological disorders. Written informed consent was obtained from all participants before the test protocol was conducted. The study and all experimental protocols were approved by the local ethics committee of the Medical Faculty of the TU Dresden and realized in accordance with the Declaration of Helsinki.

### Task

**Figure 5:**
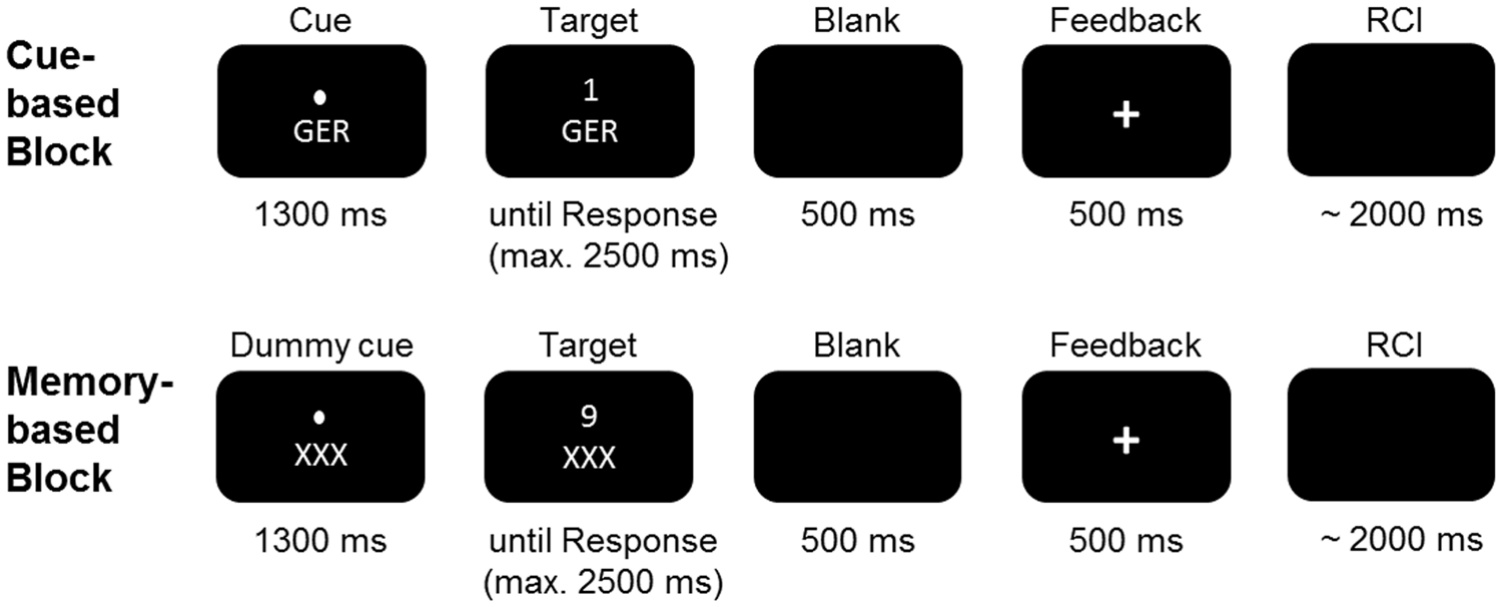
Illustration of the task switching paradigm. The cue-based task switching paradigm is shown in the upper part, the memory-based in the lower part of the figure. In the memory-based part, the subjects had to remember when to switch a rule. RCI reflects the jittered response-cue-interval.

The procedure used to examine memory-based and cue-based task switching was adapted from previous studies^7,10,63,64^. The task is shown in Fig. 5.

During task execution, participants were seated in a separate room (to prevent any disturbances or distractions) in front of a monitor and a regular keyboard. The paradigm consisted of two conditions: switching and repetition trials, which were separated into 12 experimental blocks (6 cue- and 6 memory-based blocks, with 198 trials per condition, resulting in a total of 396 trials). The cue-based block consisted of 99 switch and 99 repetition trials, the memory-based block consisted of 66 switch and 132 repetition trials. Participants were free to take a self-paced period of rest between experimental blocks. The order of blocks was counterbalanced. In all conditions, participants were instructed to respond as quickly and accurately as possible. Stimuli consisted of digits from 1–9, excluding number 5 and were presented in white font, centrally on a black computer screen (viewing distance = 56.5 cm). In the cue-based block, cues alternated randomly but with a balanced proportion of 33.3% each while the frequency of switching was set to 50%. A cue for one out of three rules (‘NUM’, ‘GER’ or ‘SG’, font size: 65) was displayed 1300 ms prior to the onset of the digits. When ‘NUM’ (short form for german word “numerisch” = engl. “numeric”) was displayed, participants were supposed to assess whether the digit was smaller or greater than 5. When ‘GER’ (for “gerade” = engl. “even”) was presented, participants had to decide whether the digit is even or odd. ‘SG’ (for “Schriftgröße”, meaning “font size”) indicated that participants had to determine whether the digit was shown in large or small font size (font size varied between 50 and 80). The cue-stimulus remained visible until the digit (target) was presented. The digit appeared at the position where the fixation point had been presented before. After target onset, the participants had to respond within 2500 ms. Otherwise, the trial was regarded as missed response. Participants had to respond using their index finger (which was either the right or the left one, based on response-hand mapping and respective experimental version), if the digit was smaller than five, had small font size or was uneven in addition they had to respond using their right index finger, if the digit was larger than five, had a large font size or was even. The responses were given on a computer keyboard using the “Ctrl” buttons. 500 ms after the response a feedback was displayed for 500 ms (“ + ” or “−” sign). After the feedback the next cue was shown. The response-cue interval (RCI) was set to ~2000 ms and included the response-feedback delay (500 ms), the feedback (500 ms) and the feedback-cue delay, which jittered.

In contrast to the cue-based block, there was a determined loop of rules which had to be kept in mind (always in the same order: {NUM, NUM, NUM, GER, GER, GER, SG, SG, SG, NUM, NUM, NUM, GER, …}). To make the two blocks more comparable, a dummy cue was presented prior to the appearance of the digits for a period of 1300 ms. This dummy cue was “XXX” (see Fig. 5). In case a participant lost count and failed to apply the correct rule in 3 consecutive trials, the regular cues (i.e., NUM, GER, or SG) replaced the dummy cue in the following 3 trials. The regular cue during the memory-based block was shown to 20 participants. In these 20 participants this happens on average once (*M* = 1.3). Just like for the cue-based block, there was a balanced proportion of each rule (33.33%), but the frequency of switching was reduced to 33.3% due to the fixed order of task rules in the memory block. It may be argued that the cue-based and the memory based differ in regularity, because there was sequence of trials in the memory-based block and random trial presentation in the cue-based block. However, in the memory-based block there needs to be a fixed trial order to invoke memory processes. If a fixed order would have been used in the cue-based block as well, this may also have induced memory-related processes that may then have confounded cue-based task switching. Before the paradigm began, participants run one practice block with 18 trials per condition.

### EEG recording and analysis

The EEG was recorded with a sampling rate of 500 Hz using a 64-channel system (BrainAmp, Brain Products Inc.) and electrode impedances were kept under 5 kΩ. Passive Ag/AgCl-electrodes (60 recording electrodes) were mounted in an elastic cap (EasyCap Inc.) and arranged in equidistant positions approximating the positions of the 10/20 system. The ground and reference electrodes were placed at coordinates theta = 58, phi = 78 and theta = 90, phi = 90, respectively. After recording, data were down-sampled to 256 Hz and filtered (band-pass filter from 0.5 to 25 Hz, with a slope of 48 dB/oct each) using the BrainVision Analyzer 2 software package (BrainProducts Inc.). Raw data was inspected manually to reject technical artifacts from the EEG. For artifacts identified in the first step we cut out as much data around an artifact as possible. Afterwards, an independent component analysis (ICA; infomax algorithm) was conducted on the un-epoched data sets to remove recurring artifacts. ICA components revealing horizontal and vertical eye movements, blinks and pulse artifacts were manually rejected. The ICA was run for all blocks combined. Prior to manual raw data inspection, noisy electrodes were deleted and topographically interpolated after all preprocessing steps.

Afterwards, the EEG data was segmented for switch and repetition trials, and for cue- and memory-based blocks separately. Only trials with correct responses were taken into account. The segments started 2000 ms before target presentation of the respective trial and ended 1500 ms after its onset. Subsequently, an automated artifact rejection procedure was conducted for all segments, with the following rejection criteria: activity below 0.5 μV in a 100 ms period and a maximal value difference of 200 μV in a 200 ms interval. If an artifact was detected in a trial at only one electrode, the entire trial was discarded. This resulted in the rejection of 23.97% of the trials during cue-based switching, 24.41% during cue-based repetition, 27.91% during memory-based switching and 29.44% during memory-based repetition. To eliminate the reference potential from the data and to re-reference the data, we applied a current source density (CSD) transformation (4 splines and 10 polynominals)^65^ which works as a spatial filter^66,67^, suppresses volume conduction and accentuates electrode sites and makes it easier to identify electrode sites that best reflect relevant neuronal activity. A baseline correction from 200 ms prior to both, cue and target onset was applied for ERP-analysis only; i.e. it was also set before the presentation of the cue and dummy-cue stimuli. Through the application of a specific cue- and target-locked baseline, we meet the needs of possible shifts of ERPs, and ensure that neural activity which might be increased due to attentive cues has no impact on the analysis of target-locked data analysis All ERP amplitudes were quantified against this baseline period at the single-subject level. Electrodes and intervals are chosen based on theoretical background, arguing where and when components show off. Accroding to^68^ the P1 wave is largest at lateral occipital electrode sites and typically onsets 60–90 ms post stimulus with a peak between 100–130 ms. The visual P1 and N1 amplitudes (which are presented in the supplementary materials) on the target stimuli, as well as the cue and dummy-cue stimuli were quantified at electrodes P7 and P8. The P1 was quantified in the time window between 90–105 ms after either cue or target stimulus presentation. According to^68^ “the P1 wave is followed by the N1 wave. N1 components that typically peak 150–200 ms post-stimulus, the lateral occipital N1 subcomponent appears to be larger when subjects are performing discrimination tasks than when they are performing detection tasks”. For this analysis, the N1 was quantified in the time window between 150–170 ms after either cue or target stimulus presentation (please refer to supplementary materials for P1 and N1 analysis). In addition to theoretical background, electrodes are also chosen by visual detection due to analysis of grand averages, separately for each condition and block.

The N2 and the P3 ERP-components on the target stimuli were quantified at electrode Cz (for the N2 ERP-component; 290–350 ms) and at electrode P4 (for the P3 ERP-component; 325–340 ms). This choice of electrode positions and time windows was validated using the methods proposed by^69^: Briefly, the above time intervals were taken and the mean amplitude within the defined search intervals was determined for each of the 60 electrode positions. This was performed only after CSD transformation of the data which emphasizes scalp topography^66^. Then, to compare each electrode against an average of all other electrodes, Bonferroni correction for multiple comparisons (critical threshold, p = 0.0007) was used. Only electrodes, which displayed significantly larger mean amplitudes (i.e., negative for the N- potentials and positive for the P-potentials) when compared to other electrodes were chosen. This procedure revealed the same electrodes as previously chosen by visual inspection.

After baseline correction, the time frequency decomposition was conducted using Morlet’s wavelets (w) in the time domain to different frequencies (f):

$${\rm{w}}({\rm{t}},\,{\rm{f}})={\rm{Aexp}}(-{{\rm{t}}}^{2}/2{\sigma }_{t}^{2})\exp (2i{\rm{\pi }}\text{ft}),$$

t = time, A = (σ_t_ √π)^−1/2^, σ_t_ = wavelet duration, and i = √−1. For analysis and TF-plots, a ratio of f_0_/σ_f_ = 5 was used, where σ_f_ is the width of the Gaussian shape in the frequency domain and f_0_ is the central frequency. The TF decomposition was applied to the single-trial data. Therefore, the total (induced) wavelet power was calculated. The analysis was conducted in the frequency range from 0.5 to 25 Hz and a central frequency at 0.5 Hz intervals was employed. For different f_0_, time and frequency resolutions (or wavelet duration and spectral bandwidth^70^; can be calculated as 2σ_t_ and 2σ_f_ respectively. σ_t_ and σ_f_ are related by the equation σ_t_ = 1/(2πσ_f_). For example, for f_0_ = 1 Hz, 2σ_t_ = 1770 ms and 2σ_f_ = 0.36 Hz; for f_0_ = 3 Hz, 2σ_t_ = 580 ms and 2σ_f_ = 1.09 Hz; for f_0_ = 5 Hz, 2σ_t_ = 350 ms and 2σ_f_ = 1.82 Hz. After TF-decomposition, the data were averaged at the single subject level to analyze the power of the alpha frequency oscillations. For statistical analysis, TF power was log_10_-transformed to normalize the distributions for statistical analyses. The power of the alpha frequency oscillations was analyzed between 8 Hz and 12 Hz (10 Hz central frequency) and quantified at electrodes P7, P8, O1, O2, PO1, PO2. These electrodes and time windows were selected on the scalp topography plots and subsequent validation of these electrode sites using the same method as used for the ERP data confirmed these electrode sites. Mean alpha band power (pooled across electrodes) was analyzed from the entire cue-target (−1300 ms – 0 ms) interval, as well as in an interval from 800 ms to 1500 ms after target presentation, in which strong alpha band activity was evident (refer Fig. 3). Importantly, due to the long and jittered response-cue interval (RCI) (~2000 ms), the analyzed time interval does not simply reflect preparatory processes of the upcoming cue-stimuli.

### Network connectivity and small world analysis

We focused on the alpha frequency band for the network connectivity analysis; i.e. on oscillations between 8 Hz and 12 Hz. The communication between all electrodes in terms of a “network” was examined by analyzing the connectivity between electrodes as the strength of association quantified by their coherence. That is, all electrodes (nodes) reflect a network and connections (edges) between nodes are defined as coherence between all possible pairs of electrodes. Therefore, only the imaginary part of the coherence spectrum for all possible pairs of nodes was calculated to effectively suppress spurious coherence driven by volume conduction^71^ For the coherence values, three different individual percentile thresholds of *P* = 85, *P* = 90, *P* = 95 was set for each subject, so only the 15, 10 or 5 percent of each individual’s highest coherence values were included in the analysis. While there are several ways to determine the threshold, for instance based on some statistical parameterization or previous observation in the literature, all of them remain arbitrary. Letting only the 10 percent of the highest values “survive” is a criteria that is a compromise or tradeoff for two problems: On the one hand, it makes sure that only electrodes with high coherence are defined as being “connected” and are included in the analysis. On the other hand, it also makes sure that enough connections between the electrodes still form a connectome and thus can be considered as a network. We therefore only reported data, where a cutoff of 10 percent of the highest values had been applied (for the results of the other thresholds, please see supplementary material). A binary 60 × 60 adjacency network matrix (because of 60 electrodes being used) was then calculated with 1 representing an un-weighted and undirected connection between any pair of electrodes and 0 representing no connection.

In order to study small world networks, the method by^72^ was used and applied to each single-subject: Starting from a one-dimensional network, where each node in the network is only connected to its *k* nearest neighbors on either side, representing a ‘regular’ network with randomness *ϐ* = 0, a ring lattice with *N* nodes of mean degree 2*k* is created. Next, with increasing randomness (*ϐ* > *0*), more connections (‘edges’) are randomly chosen to another random node. So, when *ϐ* = 0, no edges are rewired and the model returns a ring lattice. In contrast, when *ϐ* = 1, all of the edges are rewired and the ring lattice is transformed into a random network with *N* nodes and mean node degree of 2*k*. According to the Watts and Strogatz model, a network has small-world network properties if it demonstrates properties from both lattice networks, with clustered interconnectivity within groups of nodes sharing many nearest neighbors in common (high clustering coefficient, ‘C’), and properties from random networks, represented by a short geodetic distance (average path length, ‘L’) between any two nodes in the network. Thus, the balance of local segregation and global integration in neural networks (‘small worldness’) can be quantified by *C* and *L*, respectively^42^. Regular networks have a high *C* but also a very high *L*. In contrast, random networks have a low *C* and a low *L*. So, neither regular nor random networks alone can explain the small world phenomenon^72^. For each subject, average number of edges from one node to all other nodes (degree, *2k*), average shortest path length (geodetic distance, *L*_*real*_) and average clustering coefficient (*C*_*real*_) were calculated. Corresponding to each individuals degree, completely random (*ϐ* = 0) and completely regular (*ϐ* = 1) Watts Strogatz models were created and *L*_rand_ and *C*_rand_ and *C*_latt_ were computed. We analyzed all small world values (ω*)* according to^73^), who proposed a quantitative categorical definition of a small-world network in line with the definitions of the original Watts-Strogatz model^72^. In this way, the assertion, whether a network has small world properties can be tested statistically. The small-world value (SWV) formula is expressed by:

$$\omega =\frac{{L}_{rand}}{L}-\frac{C}{{C}_{latt}}$$

Small-world values of ω are restricted to the interval −1 to 1 regardless of network size. If ω is close to zero, it is considered as small world. Positive ω values represent more random properties, negative values indicates that a network has more regular or lattice-like properties.

### Statistics

The data was analyzed using repeated measures ANOVAs where the factor “block” (cue-based vs. memory-based) and the factor “condition (repetition vs. switch)” was included. For the analysis of the ERP and alpha power data, an additional factor “electrode” was included in the models. Greenhouse-Geisser correction was applied for all tests. All post-hoc tests were Bonferroni-corrected. All variables included in the ANOVAs were normal distributed as indicated by Shapiro-Wilks tests (all W > 0.65; p > 0.3). For the descriptive statistics, the mean and standard error of the mean are given.

### Data availability

The datasets generated during and/or analyzed during the current study are available from the corresponding author on reasonable request.

### Ethical standards

The authors assert that all procedures contributing to this work have been conducted in accordance with the ethical standards of the relevant national and institutional committees on human experimentation and with the Helsinki Declaration of 1975, as revised in 2008. The study and all experimental protocols were approved by the local ethics committee of the Medical Faculty of the TU Dresden.

## Electronic supplementary material

supplemental information
