# Supplementary material for: On the relevance of the alpha frequency oscillation’s small-world network architecture for cognitive flexibility
Source: Sci Rep. 2017 Oct 24;7:13910. doi: 10.1038/s41598-017-14490-x (PMC5654836; doi:10.1038/s41598-017-14490-x)
Supplement: Supplementary file 1 — supplemental information [file 41598_2017_14490_MOESM1_ESM.doc]

**Supplemental material**

**On the relevance of the alpha frequency oscillation's small-world network architecture for cognitive flexibility**

Nicole Wolff, Nicolas Zink, Ann-Kathrin Stock, Christian Beste


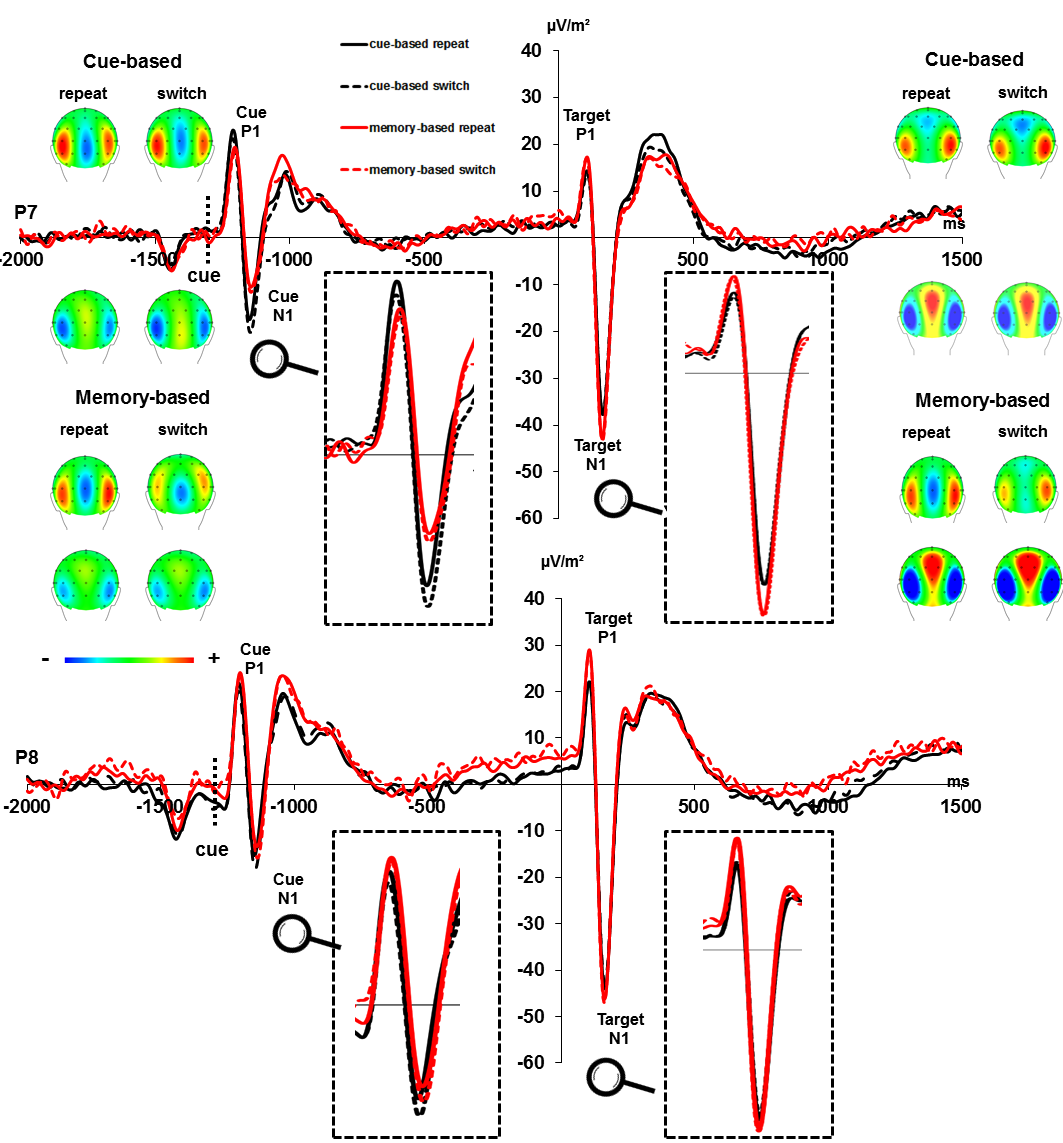


*Supplemental Figure 1*

The P1 and N1 ERP-components on the target and the cue (dummy-cue) stimuli are shown at electrodes P7 and P8. Time point zero denotes the time point of target stimulus presentation. The cue is presented 1300ms before the target. The baseline was set from -2000 ms to -1800 ms prior to target onset. Cue-based conditions are given in black lines, memory-based conditions are given in red lines. Repetitions are shown in solid lines, switches are shown in dashed lines. The scalp topography plots show the distributions of potentials across the scalp at the time point of the peak of each ERP-component. In the topography plots red colours denote positive scalp potentials, blue denotes negative scalp potentials. The plots in the top left corner of the figure show the topographies for ERP-components on the cue, the plots in the top left corner of the figure show the topographies for ERP-components on the target.

*Cue-locked ERPs*

Cue-locked P1 and N1 ERP-components were analyzed by calculating repeated measures ANOVAs using the within-subject factors “electrode” (P7, P8), “condition” (repetition, switching) and “block” (cue-based, memory-based). The data is shown in Supplemental Figure 1.

*P1*: An ANOVA on cue-locked P1 amplitudes revealed significant main effects of “electrode” (F[1,54] = 16.93, p <.001, *η^2^_p_* =.239), with larger P1 amplitudes at electrode P8 (16.25µV/m2 ± 1.27) as compared to electrode P7 (8.73µV/m2 ±1.27) and “block” (F[1,54] = 11.54, p <.001, *η^2^_p_* =.176), with increased (more positive) P1 amplitudes during memory- (13.52µV/m^2^ ±1.69) vs. cue-based (11.46µV/m^2^ ±1.45) blocks. No further effects were significant (all *p* >.225).

*N1*: The ANOVA on cue-locked N1 amplitudes revealed a significant effect of “condition” (*F*[1,54] = 37.90, *p <*.001, *η^2^_p_* =.412), showing that N1 was larger on switching
(-19.98 µV/m^2^ ± 2.32) than on repetition trials (-16.22 µV/m^2^ ± 2.09). In addition we observed a significant interaction of “electrode x block” (*F*[1,54] = 24.94, *p <*.001, *η^2^_p_* = .316). Post hoc tests, analyzing both electrodes separately revealed a significant effect on P7 (*F*[1,54] = 9.49, *p* = .003, *η^2^=* .149), showing increased (more negative) N1 amplitudes during cue- (-19.95µV/m^2^ ± 2.55) vs. memory- (-15.61µV/m^2^ ± 2.17) based blocks. Moreover on P8, we observed a significant effect of “block” as well (F[1,54] = 8.57, *p* = .005, *η^2^_p_ =* .137), showing a reversed pattern, namely increased (more negative) N1 amplitudes during memory- (-20.72µV/m^2^ ± 3.13) vs. cue- (-16.13µV/m^2^ ± 2.72) based blocks. No other main or interaction effects were significant (all *p* > .110). There were no latency effects for the P1 and N1 on cue and dummy-cue stimuli (all *p* >.5).

*Target-locked ERPs*

Target-locked ERPs of P1 and N1 were analysed by calculating mixed-effects ANOVAs using the within-subject factors “electrode” (P7, P8), “condition” (repetition, switch) and “block” (cue-based, memory-based). Target-locked P1 and N1 ERP-components are also shown in Supplemental Figure 1.

P1: An ANOVA on target-locked P1 amplitudes revealed a significant main effect of “condition” (F[1,56] = 15.79, p <.001, η2p =.226), with increased P1 amplitudes during repetition (14.57µV /m2 ± 2.05) vs. switching (12.37µV/m2 ± 2.12) trials. Further we observed a significant interaction of “electrode x block” (F[1,54] = 4.36, p = .042, η2p =.075), post hoc tests, however, testing both electrodes separately revealed no significant main effect of “block”, neither on P7 (F[1,56] = 1.48, p = .23, η2p =.027) nor on P8 (F[1,54] = .64, p = .43, η2p =.012). No further factors or interactions revealed significance (all F < 1).

N1: An ANOVA on target-locked N1 amplitudes revealed a significant main effect of “block” (F[1,54] =32.86, p <.001, η2p =.378), with increased N1 amplitudes during memory-
(-48.42 µV/m2 ± 3.98) as compared to cue-based (-41.52 µV/m2 ± 3.53) block. No further effect or interaction revealed significance (all p >.257). There were no latency effects for the P1 and N1 on cue and dummy-cue stimuli (all p >.5).

*Analysis of the alpha band small world network architecture*

*
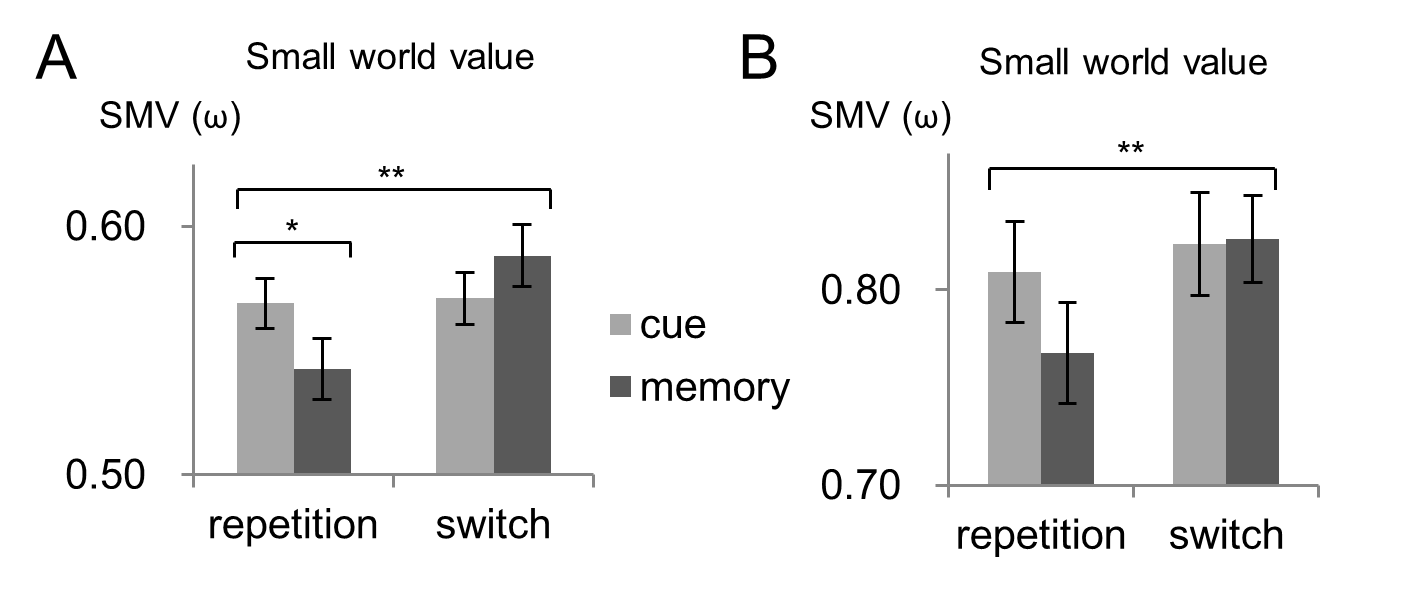
*

*Supplemental Figure 2*

Small world values (SWVs; ω) (y-axis) for cue and memory-based trials in the repetition and the switching condition. The mean and standard error of the mean are given. (A) Small world values for coherence threshold of top 15%. (B) Small world values for coherence threshold of top 5%.

*Post-Target processes of alpha-band oscillations (800 – 1500ms )with coherence threshold of top 15%.*

Results are shown in Supplemental Figure 2A. The analysis of the SWV of alpha frequency activity in the time range from 800ms to 1500ms after target stimulus revealed the following effects: A main effect "condition" (*F*[1,54] = 9.05; *p* = .004; *η^2^_p_* = .139) showed that the SWV was smaller during repetition (0.56 ± 0.009) than switching trials (0.59 ± 0.009). There was also an interaction "block x condition" (*F*[1,54] = 7.48; *p* = .008; *η^2^_p_* = .118). Post-hoc tests showed that for switching trials no difference between the memory-based and the cue-based block was evident (*t*[54] = 1.24; *p* > .2). In the repetition trials, the SWV was larger in the cue-based condition (0.57 ± 0.08) than in the memory-based condition (0.54 ± 0.09) (*t*[56] = 2.17; *p* = .034). Furthermore, there was no difference in the SMV between repetition and switch trials in the cue based block (t[54] = -.185; *p* = .85), whereas in the memory based block the switch trials (0.59 ± 0.09) had a larger SMV than the repetition trials (0.54 ± 0.09).

*Post-Target processes of alpha-band oscillations (800 – 1500ms ) with coherence threshold of top 5%:*

Results are shown in Supplemental Figure 2B. The analysis of the SWV of alpha frequency activity in the time range from 800ms to 1500ms after target stimulus revealed a main effect "condition" (*F*[1,54] = 4.96; *p* = .03; *η^2^_p_* = .081) with smaller SWV during repetition (0.78 ± 0.023) than switching trials (0.83 ± 0.021). All other effects were not significant (all *F*≤2.34; *P*≥0.132).
